# Two alternative methods for the retrieval of somatic cell populations from the mouse ovary

**DOI:** 10.1093/molehr/gaab033

**Published:** 2021-05-10

**Authors:** E R Frost, E A Ford, G Taylor, S Boeing, E L Beckett, S D Roman, R Lovell-Badge, E A McLaughlin, J M Sutherland

**Affiliations:** 1 Priority Research Centre for Reproductive Science, Schools of Biomedical Science & Pharmacy and Environmental & Life Sciences, University of Newcastle, Callaghan, NSW, Australia; 2 Hunter Medical Research Institute, New Lambton Heights, NSW, Australia; 3 Stem Cell Biology and Developmental Genetics Lab, The Francis Crick Institute, London, UK; 4 Bioinformatics and Biostatistics Facility, The Francis Crick Institute, London, UK; 5 Scientific Computing—Digital Development Team, The Francis Crick Institute, London, UK; 6 School of Environmental and Life Sciences, Faculty of Science, University of Newcastle, Callaghan, NSW, Australia; 7 Priority Research Centre for Drug Development, University of Newcastle, Callaghan, NSW, Australia; 8 School of Science, Western Sydney University, Penrith, NSW, Australia; 9 School of Biological Sciences, Faculty of Science, University of Auckland, Auckland, New Zealand

**Keywords:** granulosa cells, granulosa cells, ovarian follicle, follicle development, dissociation

## Abstract

Many modern techniques employed to uncover the molecular fundamentals underlying biological processes require dissociated cells as their starting point/substrate. Investigations into ovarian endocrinology or folliculogenesis, therefore, necessitate robust protocols for dissociating the ovary into its constituent cell populations. While in the mouse, methods to obtain individual, mature follicles are well-established, the separation and isolation of single cells of all types from early mouse follicles, including somatic cells, has been more challenging. Herein we present two methods for the isolation of somatic cells in the ovary. These methods are suitable for a range of applications relating to the study of folliculogenesis and mouse ovarian development. First, an enzymatic dissociation utilising collagenase and a temporary, primary cell culture step using neonatal mouse ovaries which yields large quantities of granulosa cells from primordial, activating, and primary follicles. Second, a rapid papain dissociation resulting in a high viability single cell suspension of ovarian somatic cells in less than an hour, which can be applied from embryonic to adult ovarian samples. Collectively these protocols can be applied to a broad array of investigations with unique advantages and benefits pertaining to both.

## Introduction

**Figure 1.**
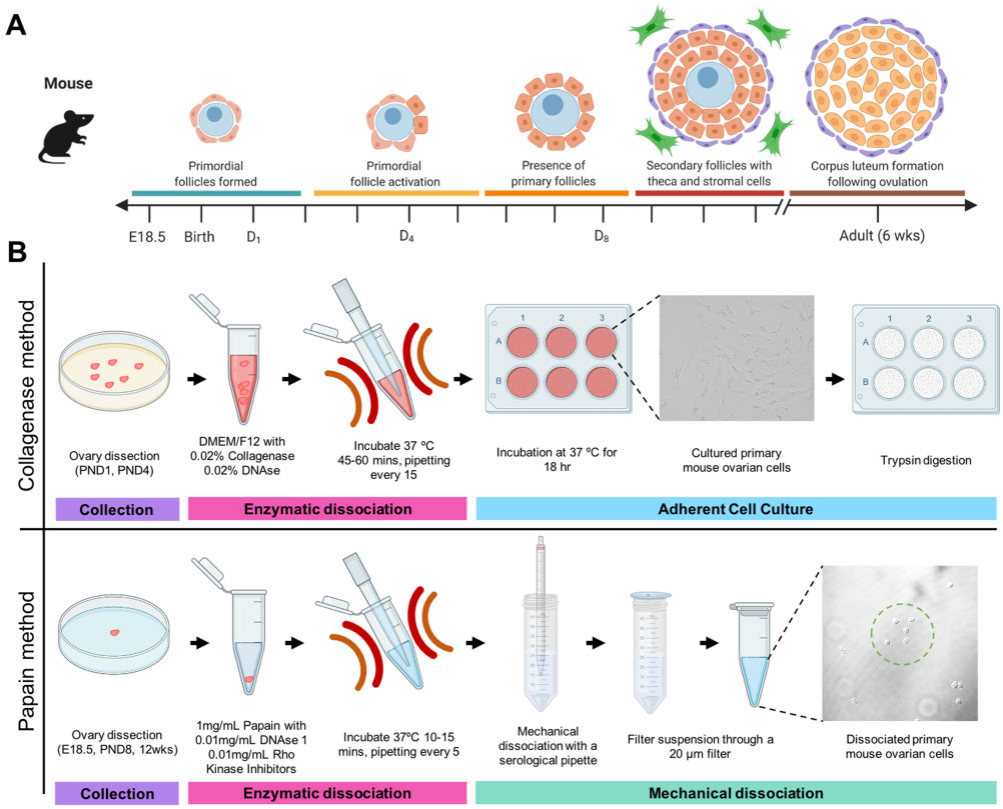
**Schematic diagram showing the timeline of ovarian follicle development in the mouse and the two dissociation protocols to retrieve somatic cells from these ovarian timepoints**. (**A**) Relative timeline showing the key stages of folliculogenesis in embryonic mice (E = embryonic day) and post-natal mice (PND = days post birth). Germ cell cysts, also known as nests, breakdown to form primordial follicles (teal bar), containing an oocyte (blue) and pre-granulosa cells (orange), then the initial wave of primordial follicle activation occurs (yellow bar) which sets the ovarian reserve, leaving a proportion of primary follicles (orange bar) present in the ovary. Primary follicles then continue through folliculogenesis to form secondary follicles (red bar), which are surrounded by theca cells (purple) and stromal cells (green). Once the mice become sexually mature, around 6 weeks of age, corpus luteum structures form following ovulation, and the granulosa cells become luteinised and produce hormones (brown bar). Figure adapted from (Sarraj and Drummond, 2012), figure made using Biorender.com. (**B**) Schematic diagrams of the key steps in the collagenase and papain methods. Both methods involve collection steps (purple bar) and enzymatic dissociation steps (pink bar). The collagenase method uses differential cell adhesion in culture to select for granulosa cells (teal bar). In contract, the papain method using mechanical dissociation and filtration to enrich for somatic cells (blue bar). Figure made using Biorender.com.

The mammalian ovary undergoes dynamic changes during early embryonic and postnatal development to ensure fertility during the female reproductive lifespan. To facilitate these changes, the ovary contains multiple, heterogenous cell populations. The germ cell populations transition from primordial germ cells to oocytes subject to meiotic maturation. The somatic cell populations include pre-granulosa cells, granulosa cells, theca cell progenitors, theca cells and stromal cells, as well as luteal cell types after ovulation. In particular, granulosa cells have a range of different roles in the mature ovary, from the dormant, undifferentiated pregranulosa cell in the primordial follicle, to the steroidogenic, mitotic mural granulosa cell of the antral follicle (Matsuda *et al.*, 2012). Detailed characterisation of these cell populations has been achieved in the adult mouse, once these cells have ceased differentiating (Liu *et al.*, 2015). Less work has been performed in the developing ovary, where various important developmental processes and cell fate decisions occur. During early ovary development, granulosa cells represent the majority somatic cell type in the ovary. During gestation in the mouse, the granulosa cells in particular drive the process of sex determination, and then facilitate germ cell nest breakdown, primordial follicle formation and primordial follicle activation (Fig. 1) (Fu *et al.*, 2018; Lv *et al.*, 2019; Stevant and Nef, 2019). To support these numerous developmental pathways, there is heterogeneity within the granulosa cell population (Rastetter *et al.*, 2014; Niu and Spradling, 2020; Wang *et al.*, 2020). For example, two populations of primordial follicles are formed within the ovary, distinguished by their location in the ovary and the expression of either Forkhead box L2 (FOXL2) or Leucine Rich Repeat Containing G Protein-Coupled Receptor 5 (LGR5) in the granulosa cells (Rastetter *et al.*, 2014; Niu and Spradling, 2020). This heterogeneity is complex to study in the ovary, as the granulosa cells remain directly connected to the germ cells throughout development. New techniques and methodologies, like single-cell RNA-sequencing and CRISPR-CAS9 gene editing show huge potential in understanding the specific granulosa cell-regulated mechanisms throughout development.

There are several technical challenges to overcome relating to obtaining large quantities of viable granulosa cells for analysis, including the small size of the ovary during embryonic and postnatal development. Further technical challenges include the tight cellular association between granulosa cells and the oocyte and the lack of knowledge surrounding factors that distinguish the various pre-granulosa and granulosa cell populations. Common methods of tissue analysis like histological sections, or even whole tissue clearing studies (now achieved in ovaries; Feng *et al.*, 2017) observe the cells at a static point in time and always in the context of the follicular structure. Additionally, the isolation of follicles from the ovary, and the use of laser capture microdissection (as in Oktay *et al.*, 1997; Dolmans *et al.*, 2006; Bonnet *et al.*, 2011; Ernst *et al.*, 2017, 2018; Steffensen *et al.*, 2018) require significant and laborious manual effort into either classifying whole follicles by their diameter using pre-defined criteria (such as Pedersen and Peters, 1968), or by visually identifying each cell of interest to be isolated. All these methods require the tissue to be fixed first prior to isolation and are unable to yield a live cell population.

In other heterogenous cell populations, the isolation of a particular subset of cells is typically performed via fluorescence-activated cell sorting (FACS). However, there are currently no reliable cell surface markers or mice transgenic for fluorescent reporters such as green fluorescent protein (GFP) that are capable of distinguishing the primary follicle granulosa cells. Most recent protocols published on the mouse ovary detail the dissociation of ovarian tissue to retrieve oocytes or oogonial stem cells (OSCs) (Woods and Tilly, 2013; Navaroli *et al.*, 2016; Zarate-Garcia *et al.*, 2016; Clarkson *et al.*, 2018). Thus, there is a demonstrated need for a method of separating granulosa cells from the follicle that requires less manual input achieving a sufficient yield for standard and advanced laboratory procedures, such as extraction of protein, DNA, and RNA, immunocytochemistry, discovery proteomics and mass spectrometry, and RNA sequencing. Such a technique should keep cells alive, ideally within minimal experimental steps and using no prior fixation or staining to maintain original cellular expression and other physiological characteristics.

A common solution involves the enzymatic dissociation of the ovarian tissue. Enzymatic dissociation represents an established procedure yet the primary outcomes of studies utilising this technique have largely centred on the isolation of whole follicles rather than individual cells (Dolmans *et al.*, 2006), or isolating granulosa cells from mature ovaries (Morbeck *et al.*, 1993). Enzymatic dissociation typically relies on the breakdown of the outer structure of the ovary (usually via collagenase, liberase or papain), to release the cells or follicular structures, followed by a temporary culture step to allow the granulosa cells to adhere to the surface while oocytes and debris remain suspended in the media. Collagenase is a more efficient enzyme for isolating individual cells, because it is known to break apart theca cells and disrupt follicular structure (Newton *et al.*, 1999). Enzymatic dissociation methods, combined with mechanical dissociation, also allow for the preparation of a single cell suspension from ovarian tissue, necessary for the purpose of single-cell RNA-sequencing (scRNAseq). ScRNAseq requires sufficient tissue dissociation and high cell viability, to ensure the sequencing is of high quality and not contaminated by RNA from dying cells. Several recent studies have performed ovarian dissociation at various timepoints for sequencing studies, listed in Table I. All listed studies have different dissociation conditions, introducing variability between the data obtained from these studies. In this manuscript, we optimise the papain enzyme for obtaining suspensions of single cells from the mouse ovary. Papain is a cysteine protease and is frequently used in the dissociation of brain tissue (Carter *et al.*, 2018; Ho *et al.*, 2018; Yousef *et al.*, 2018). It has been shown that extended dissociation in enzymatic solutions may reduce cell viability (Woods and Tilly, 2013), so short enzymatic dissociation coupled with mechanical dissociation is generally preferred to limit transcriptomic changes before scRNAseq (Nguyen *et al.*, 2018). The papain dissociation protocol was adapted from three dissociation protocols commonly used for small tissue dissociations (Woods and Tilly, 2013; Williams *et al.*, 2019; Weinberger *et al.*, 2020).

**Table I:** Comparison of published protocols used to dissociate mouse ovarian tissue.

Year	Timepoint	Mouse	Mincing of tissue?	Enzymes used	Cell types isolated	Filtration used?	Enzyme dissociation time	Reference
2012	Adult (8wk)	Wild-type C57BL6	Mince using a scalpel	800 U/ml Collagenase 0.001 mg/ml DNAse 1 0.05% trypsin-EDTA	All ovarian cells	70 µm	15min	White *et al*. (2012)
2013	Adult (6–8 weeks)	Wild-type—no specific strain	Mince using a scalpel	800 U/ml Collagenase 0.001 mg/ml DNAse 1	Oogonial stem cells	100 µm	30 min	Woods and Tilly, (2013)
2019	E10.5—E13.5, E16.5, PND6	Tg (Nr5a1-GFP) CD1 mice	No	Trypsin 0.05% EDTA	Somatic cells	40 µm	10 min	Stévant *et al*. (2019)
2019	PND2 and PND6	Wild-type F1 mice	No	0.05% Trypsin 0.53 mM EDTA 0.02% DNAse 1	All ovarian cells	No	30 min	Lo *et al*. (2019)
2020	E11.5, E12.5, E14.5, E16.5, E18.5, PND1 and PND5	Wild-type—no specific strain	No	0.25% Trypsin	All ovarian cells	70 µm then 30 µm	E11 and 12 20 min E14 and 16 40 min E18 60 min P1 and 5 80 min	Niu and Spradling, (2020)
2020	E16.5, P0, P6	Wild-type C57BL6	Cut into small pieces	0.25% Trypsin 2 mg/ml Collagenase	All ovarian cells	40 µm	6–8 min	Wang *et al.* (2020)
2021	E12.5, E13.5, E16.5, E18.5	Tg (Nr5a1-GFP) CD1 mice	No	Trypsin 0.05% EDTA	All ovarian cells	70 µm	15 min	Mayère *et al*. (2021)

Here we present two methods for the dissociation of the mouse ovary, which are appropriate for the main molecular biology techniques used to characterise and investigate the somatic cell populations present across ovary development. The collagenase dissociation method yields a largely pure granulosa cell population from the early postnatal mouse ovary and is suitable for primary cell culture and techniques requiring a large amount of sample. Due to the culture step, this protocol is primarily recommended for downstream experiments including *in vitro* genetic manipulation studies, like CRISPR and transfection, as well as *in vitro* treatment experiments for the testing of inhibitors and toxicological compounds in granulosa cells (Fig. 1). In contrast, the papain dissociation protocol captures all the somatic cell types from the embryonic through to adult ovary and is thus a useful protocol for sequencing studies to characterise gene expression in multiple cell linages across a diverse developmental range. The papain protocol allows for consistency in studies where multiple ovarian timepoints are to be studied, and experiments can be performed with one ovary per replicate, limiting the number of mice required for experimental techniques. The papain dissociation protocol was optimised for high-throughput experiments, including single-cell and bulk RNA-sequencing, so it is also a suitable protocol for the validation of novel genes from these experiments, including quantitative PCR and immunocytochemistry (Fig. 1). In this study detailing the two dissociation methods, we characterise the cells retrieved from both methods and show that key granulosa genes are expressed, and cell identity is maintained using both methods. These methods allow for further investigations into granulosa and somatic cell function, and the identification of potential markers to identify the various sub-classes of granulosa cells throughout development.

## Materials and methods: Part 1

### Reagents for collagenase dissociation method

Antiseptic solution (i.e. iodine solution; Perrigo Australia, Belrose, NSW, Australia, RIO00802F, used in this protocol)

Ascorbic acid (Sigma, St. Louis, MO, USA, A0278) made up to 5 mg/ml in MilliQ water

Bovine serum albumen (BSA) (Sigma, A7906)

Collagenase type II (Sigma, C6885)

Dulbecco's Modified Essential Medium/Ham's F-12 Medium (DMEM/F12) (Sigma, D6421)

DNase I (Roche, Basel, Switzerland, 11284932001)

Fetal bovine serum (FBS) (Life Technologies, Carlsbad, CA, USA, 10099141)

Hank’s Balanced Salt Solution (HBSS) (Life Technologies, 14025-092)

Hepes solution, 1 M (Thermofisher, Waltham, MA, USA, SH30237.01)

Insulin-Transferrin-Selenium (ITS -G) (Life Technologies, 41400-045)

Leibovitz medium (L-15) (Life Technologies, 21083-027)

L-Glutamine, 200 mM (Thermofisher, 25030-81)

Penicillin-streptomycin, 10,000 U/ml (Thermofisher, 15140-122)

Trypan blue (Invitrogen, Carlsbad, CA, USA, T10282)

Trypsin/Ethylenediaminetetraacetic acid (EDTA) (Sigma, T4049)

### Equipment for collagenase dissociation method

The following list includes necessary equipment and consumables are presented. The product details of the consumables that were utilised in the collection of this data are presented for transparency, but similar products should be sufficient to replicate these procedures.

Benchtop centrifuge compatible with 15 ml tubes and capable of 800*g*

Biosafety cabinet

Cell counter

Cell culture plate, 6-well (Greiner Bio-one, Kremsmünster, Austria, Cellstar)

Centrifuge tubes, 15 mL (Greiner Bio-one, 188261)

Filter, 0.22 µm size (Sarstedt, Newton, NC, USA, 83.1826.001)

Haemocytometer

Heated microscope stage

Incubator with 5% CO_2_ in air

Microcentrifuge

Microcentrifuge tube, 1.5 mL (Sarstedt, 72.690.001)

Round bottom polypropylene tube with cap for aeration (BD, Franklin Lakes, NJ, USA, 352059)

Serological pipettes, filtered, 10 mL and 25 mL volumes (from Sarstedt)

Small petri dish, IVF petri dish (Thermofisher, 150255) used in this protocol

Stereo microscope

Water bath

*Optional:* Cell culture plates, 24 or 48-well

*Optional:* Glass pipette tips, 20 μl, 200 μl, 1000 μl volumes

*Optional:* Glass serological pipettes, 1 ml, 10 ml volumes

*Optional:* Paraformaldehyde diluted to 4% (w/v) in phospho-buffered saline solution

### Solutions for collagenase dissociation method

Dissociation working solution: prepare 1 ml of DMEM/F12 in a 1.5 ml microfuge tube and heat to 37°C. Dissolve 0.02% (2 mg) Collagenase II, and 0.02% (2 mg) DNase I. To be made fresh each time.

Hank’s solution: under sterile conditions, prepare a 50 ml container, adding 125 mg of BSA, 0.5 ml 1 M Hepes (pH 8.0), and bring to 50 ml with HBSS. Filter sterilise and store at 4°C until use.

Ovary culture media: under sterile conditions, in a 50 ml container, add 25 mg BSA, 1 ml penicillin–streptomycin, 1.25 ml FBS, 125 µl ITS-G, and 78 µl l-glutamine. Make the solution up to 25 ml with DMEM/F12 media. To be stored at 4°C and heated to 37°C before use.

### Protocol for collagenase dissociation method

#### Isolation of granulosa cells (Day 1)

1. For collecting ovaries of sizes up to 20 mg (approximately 1 adult ovary, or 4–8 neonatal ovaries which are the subject of this protocol), prior to specimen collection, prepare a small petri dish containing L-15 media, supplemented with 1% FBS and allow to heat to 37°C.

*Note*: where >20 mg of the specimen is to be dissociated, it is optimal to set up multiple dissociations in parallel rather than to increase the reaction sizes in a single dish or tube.

2. Add harvested ovary (or ovaries) to the dish (see Appendix 1 for ovary dissection details). Transfer the dish to a heated stage (at 37°C) under a stereo microscope, remove the ovarian bursa and oviduct keeping only ovary tissue for dissociation.

3. Prepare 1 ml of the dissociation working solution (pre-warmed to 37°C in a water bath) in a round-bottom tube (with a loose-fitting cap for gas exchange), transferring the tissue from L-15 into the dissociation solution.

4. Incubate at 37°C in 5% CO_2_ for 45–60 min depending on the size and degradation level of the tissue, until the tissue has released a majority of its contents. Occasionally some clumps of tissue remain, as fibrous tissue does not completely disintegrate. Pipette the solution using a P200 pipette tip that has been pre-coated with Hank’s solution every 15 min to mechanically facilitate the dissociation of the tissue.

*Note:* once the ovary has started to break down, all pipette tips for subsequent steps should be pre-coated with Hank’s solution, where the BSA helps to prevent cells adhering to pipette walls. Glass pipette tips are an optional addition and when pre-coated in Hank’s solution, further reduce cell loss.

5. Transfer the solution to a microcentrifuge tube, using pre-coated pipette tips, and centrifuge for 1500*g* for 3 min.

6. Remove the supernatant and add Trypsin-EDTA. Incubate at 37°C for 20 min (for tissue sizes <10 mg, incubate for 15 min).

7. Inactivate Trypsin by the addition of FCS and mix the solution by gently pipetting. Then centrifuge solution at 1500*g* for 3 min.

8. Under sterile conditions in a biosafety cabinet to avoid contamination, remove the supernatant and resuspend the pellet in 100 μl of ovary culture media pre-heated to 37°C.

9. Remove an aliquot of cells and dilute 1:1 with Trypan Blue for cell count and viability measurements using haemocytometer or other cell counting method.

*Note:* cell count on Day 1 is a homogenised mix of the assortment of cell types in the ovary and cell count numbers may not necessarily reflect the number of cells obtained on Day 2.

When the purpose of the dissociation is to extract protein or RNA, proceed with Step 10 and Day 2, or see protocol: ‘preparation for immunocytochemistry’.

10. Where cell count is ≤3 million in total, prepare a 6-well plate for primary culture by adding 2 ml of ovary culture media per well, with 10 μl ascorbic acid. Add up to 50 000 cells per cm^2^ (or around 500 000 cells per well of a 6-well plate) then place in 37°C incubator for 18 h. Scale up reagents as necessary where cell count exceeds 3 million by adding cells to a larger flask or by including a second 6-well plate.

#### Day 2 of collagenase dissociation method

1. In a biosafety cabinet and using a serological pipette, remove the media from the plate (granulosa cells adhere to the base of the plate) and dispose of in antiseptic solution.

*Note:* avoid disturbing the base or side of the plate as much as possible so as to not disrupt the granulosa cells.

2. Rinse adhered cells with 2 ml HBSS per well by gently dropping the liquid and swirling the plate for a few rotations to thoroughly coat the surface of the wells. Withdraw the wash liquid and discard in antiseptic solution.

*Note:* for all steps where liquid is being exchanged on the plate of cells, do not let the cells dry out. Proceed through these steps quickly, and cover with the lid to reduce drying when unattended, even momentarily.

**Figure 2.**
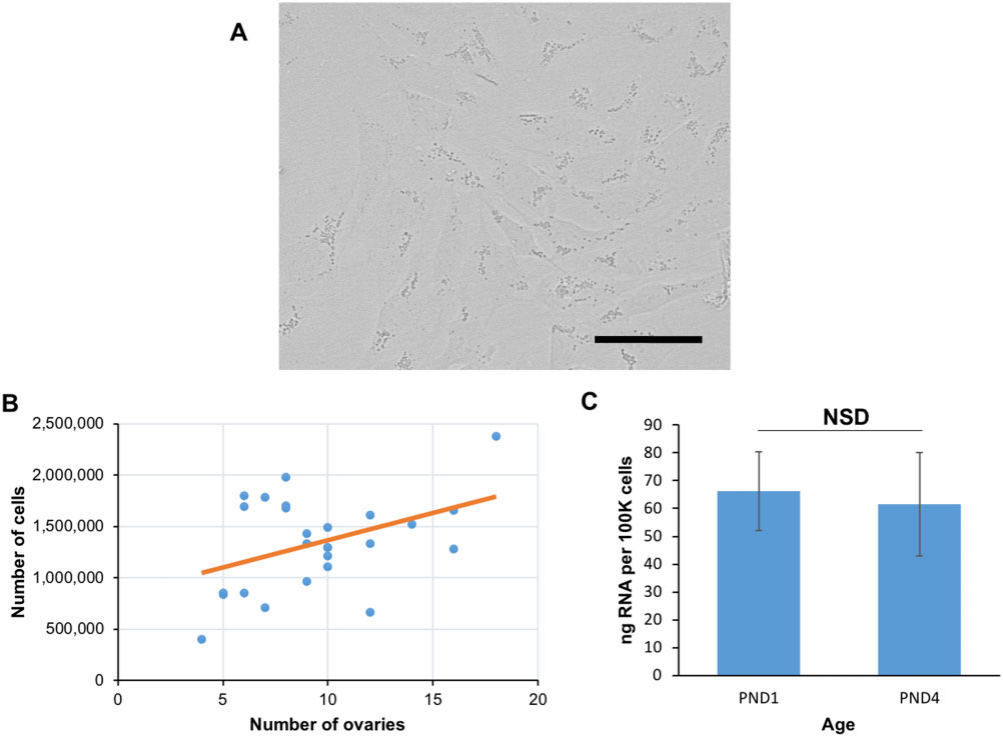
**Granulosa cell characteristics after collagenase dissociation**. (**A**) Bright field images of granulosa cells adhering to plate surface following enzymatic dissociation and overnight culture. Images taken at 40× magnification, scale bar equivalent to 50 μm, (**B**) granulosa cell yield by number of ovaries dissociated in a single replicate with linear fit, and (**C**) RNA yield (ng per 100 000 cells) by age of ovaries dissociated. Data presented is from 25 dissociation replicates, 11 utilising PND1 ovaries, and 14 replicates of PND4 ovaries. NSD, no significant difference; PND, days post birth.

3. Pre-treat the cells with a short trypsin digestion by adding 1 ml of trypsin-EDTA to each well (again, gently dropping the liquid to avoid disruption) and swirling the plate for 10–20 s. Then, withdraw and discard the trypsin.

4. Add 1 ml of trypsin to each well of the plate, and incubate at 37°C for 5 min, or until cell projections are removed from the surface of the plate (Fig. 2).

5. Inactivate by adding FBS to the trypsin at a ratio of 1:5 and mix mechanically by pipetting.

6. Collect the cells by repeatedly pipetting the trypsin/FBS solution over the surface of the plate to lift any remaining adherent cells and transfer the liquid into a 15 ml centrifuge tube.

*Note:* in a 6-well plate, a high proportion of cells adhere along the edge of the wells and the fine tip of a p20 pipette tip can lift these cells with a concentrated ejection of liquid.

7. Do a final wash of the plate with 1 ml of HBSS per well, swirling the liquid and pipetting over the surface of the plate, then add the liquid to the 15 ml cell suspension.

*Note:* using a microscope on bright field setting, inspect the plate between washes for any remaining cells. An additional wash of 1 ml HBSS can be performed if necessary.

8. Spin the tube containing cells in a benchtop centrifuge for 5 min at 800*g*.

*Note:* the final steps may be performed outside of the biosafety cabinet. For final steps, it is crucial that pipette tips coming into contact with cells are pre-coated with Hank’s solution to avoid cell loss in the plastic tips. Additionally, the pellet may be difficult to visualise by eye, so ensure the supernatant is carefully removed by pipetting from the opposing side of the tube to the pellet.

9. Discard the supernatant and resuspend the pellet in 1 ml of HBSS to wash the cells (transferring to a 1.5 ml tube for efficiency). Spin the cells in a microfuge for 5 min at 1200*g*.

10. Remove the supernatant and repeat the washes by resuspending in HBSS and centrifugation for a total of three washes.

11. Discard the supernatant of the final wash and resuspend the cells in a small volume of HBSS for a final cell count as described in Day 1, Step 9.

12. Centrifuge cells at 1200*g* for 5 min and proceed with preparation for either protein or RNA extraction. Alternatively, cell pellets may be stored as bulk samples at −80°C for later use.

#### Preparation for immunocytochemistry

1. Working in a biosafety cabinet, prepare a plate or series of plates (24- or 48-well depending on the number of replicates or cell numbers required) for primary culture by adding an appropriate volume of ovary culture media per well (500 μl for 24-well, and 200 μl for 48-well), with a volume of (5 mg/ml) ascorbic acid totalling 0.5% of the solution. Add up to 50 000 live cells per cm^2^, then place in 37°C incubator for 18 h.

2. Continuing in a biosafety cabinet after incubation, spike the growth media with 4% paraformaldehyde (at a ratio of 1 part 4% paraformaldehyde to 4 parts media) and incubate at room temperature for 2 min.

3. Discard liquid into antiseptic solution and replace with an equivalent volume of 4% paraformaldehyde for 10 min at room temperature to fix cells.

*Note:* for all steps where liquid is being exchanged on the plate of cells, do not let the cells dry out. Proceed through these steps quickly, and cover with the lid to reduce drying when unattended. Additionally, avoid disturbing the base or side of the plate as much as possible so as to not disrupt the granulosa cells.

4. Discard the leftover paraformaldehyde and rinse cells with HBSS to remove excess fixative by gently dropping the liquid and swirling the plate for a few rotations to thoroughly coat the surface of each well. Withdraw the wash liquid and discard in antiseptic solution. Repeat for a second wash.

5. Cover each well with HBSS, seal the plate with the lid. Can be stored for up to 3 months at 5°C.

## Materials and methods: Part 2

### Reagents for papain dissociation method

Bovine serum albumin (BSA) (Sigma, A7906)

DNase I (Merck, Kenilworth, NJ, USA, 10104159001)

10 x Hank’s Balanced Salt Solution (HBSS) (Thermofisher, 14185052)

Papain (Roche, 10108014001)

1 x Phosphate Buffered Saline (PBS) (Sigma, P4417)

Rho associated kinase inhibitor Y-27632 (Abmole Bioscience, Houston, TX, USA, M1817)

1 M TRIS pH 8.0 (Thermofisher, AM9855G)

Trypan Blue (Invitrogen, T10282)

### Equipment papain dissociation method

Low speed centrifuge with tissue culture rotors (compatible with 15 ml and 50 ml Falcon tubes)

Biosafety tissue culture hood

Microcentrifuge

Stereo microscope

37°C water bath

IVF Petri Dish, 35 mm, with Lid (Thermofisher, 150255)

3-Well glass plate dish

Syringe and filter, 0.22 μm size

15 ml Falcon tubes

50 ml Falcon tubes

20 μm Pre-separation Filters (Miltenyi Biotec, Bergisch Gladbach, Germany, 130-101-812)

10 mL Pyrex^®^ glass disposable serological pipettes (Merck, CLS707710N)

1.5 mL DNA LoBind tubes (Eppendorf, Framingham, MA, USA, EP0030108051)

20 μL Axygen Low binding filter tips (Thermofisher, 14-222-799)

200 μL Axygen Low binding filter tips (Thermofisher, 14-222-777)

1000 μL Axygen Low binding filter tips (Thermofisher, 14-222-765)

12-well Thermo Scientific™ PTFE Diagnostic Slides (Thermofisher, 10028210)

*Optional:* Countess II Cell Counter (Invitrogen, AMQAX1000)*Optional:* Countess™ Cell Counting Chamber Slides (Invitrogen, C10312)*Optional:* Haemocytometer

### Solutions for papain dissociation method

Hank’s solution: In a 50 ml Falcon tube, add 5 ml of 10 × HBSS, 125 mg of BSA and 0.5 ml of 1 M TRIS pH 8.0. Bring the solution to 50 ml with distilled water, and filter sterilise using a 0.22 μm filter. To be made fresh each time.

Dissociation solution: Prepare 900 µl of Hank’s solution in a 1.5 ml tube. Add 100 µl of papain, 1 µl of DNAse I and 1 µl of Rho associated kinase inhibitor to the 1.5 ml containing Hank’s solution. Prewarm dissociation solution at 37°C until tissue is ready for dissociation.

### Ethics

Australia: C57BL/6J mice were supplied by the University of Newcastle Animal Services Unit under ethics approval number A-2018-803. All experiments involving the use of animals were conducted in accordance with the Institutes’ Animal Care and Ethics Committee guidelines in accordance with Australian NHMRC Guidelines on Ethics in Animal Experimentation.

UK: All experiments carried out on mice were approved under the UK Animal (Scientific procedures) Act (Project licence 80/2405).

### Protocol for papain dissociation method

This protocol was developed for the isolation of somatic cells from one neonatal mouse ovary across a range of time points (embryonic day (E)18.5, post-natal day (PND) 8 and 12 weeks (WKS)).

1. Pre-warm a water bath to 37°C.

2. Prepare a small petri dish containing 1 × PBS.

3. Pre-warm 500 μl of papain dissociation in a 1.5 ml LoBind microcentrifuge tube.

4. Carefully dissect the ovaries from the female mouse, by dissecting with the uterus and ovarian bursa.

5. Add harvested ovary to the PBS dish. Transfer the dish to the stereo microscope, gently remove the ovarian bursa and oviduct, keeping the ovary tissue for dissociation.

If collecting embryonic or early post-natal ovaries, proceed straight to Step 11. If collecting somatic cells from an adult ovary, proceed to Step 6.

6. Place 500 μl of papain/HBSS/DNase 1/Rho inhibitors dissociation solution into a glass plate.

7. Once ovary is cleaned of fat in PBS, transfer ovary to the well containing 500 μl papain.

8. Mince the ovary using mincing scissors or a scalpel, and transfer liquid containing cells to 1.5 ml Eppendorf tube, max 1 ovary per tube.

9. Wash the glass well with 500 μl of papain dissociation solution and add to 1.5 ml Eppendorf tube (should total 1 ml of dissociation solution).

10. Once the ovary is minced, transfer the ovary tissue into the LoBind microcentrifuge tube containing the papain dissociation solution.

*Note:* once the ovary is dissociated, all pipette tips for subsequent steps should be pre-coated with Hank’s solution to prevent cells adhering to pipette walls. Pre-coating involves pipetting the Hank’s solution up and down with the pipette tip several times.

11. Incubate the tissue in the papain dissociation solution for 10 min in the 37°C water bath.

12. Homogenise the tissue in the papain dissociation solution after 5 min, with a P200 tip that has been pre-coated with Hank’s solution. Gently pipette up and down 10 times against the wall of the tube to disrupt the tissue.

13. Stop the reaction by adding the dissociated ovarian cells to 4 ml of Hank’s solution in a 50 ml falcon tube. Rinse the 1.5 ml microcentrifuge tube with another 1 ml of Hank’s solution and re-unite with the reminder of the cells in the falcon tube.

14. Pre-coat a 10 ml glass serological pipette with Hank’s solution.

15. Use the 10 ml glass serological pipette to pipette the cells up and down 10–15 times against the bottom of the 50 ml tube containing the homogenised material.

16. In a low-speed centrifuge, spin down the dissociated cells at 500*g* for 5 min at room temperature.

17. Remove the supernatant using a glass pipette and dispose of the waste. Resuspend the cells in 4 ml of Hank’s solution (glass pipettes should also be pre-coated with Hank’s solution).

18. Pass the dissociated cells through a 20 mm filter, placed into a clean 15 ml tube. Rinse the 50 ml falcon tube with 1 ml of Hank’s solution and add this to the filter.

19. Once the liquid has passed through the filter, remove the filter and discard, and place the lid on the 15 ml tube.

20. Centrifuge the cell suspension at 500*g* for 5 min.

21. Remove the supernatant using a glass pipette and resuspend the cells in 1 ml of Hank’s solution. Transfer the 1 ml of cell suspension to a 1.5 ml LoBind Eppendorf tube and take a 10 μl aliquot for cell counting.

22. Centrifuge the 1 ml cell suspension at 500*g* for 5 min.

23. Perform a cell and viability count with the 10 μl aliquot of cells, by diluting the aliquot 1:1 with the live/dead stain Trypan Blue and pipetting the solution into the Cell Chamber slides for analysis by the Countess II. A haemocytometer can also be used for a count and viability measurement.

24. Once the centrifugation is complete, remove the supernatant using a P1000 pipette. Cell pellets can be stored at −80°C, prepared immediately for protein or RNA extraction or fixed for immunocytochemistry (protocol below).

#### Preparation for immunocytochemistry

1. Following the cell dissociation, resuspend the cells in 4% PFA in PBS and fix cells for 20 min at room temperature with gentle nutation.

2. Spin cells down at 800 rpm for 5 min to pellet.

3. Remove the supernatant using a P1000 pipette and resuspend the cells in PBS.

4. Spin cells down at 800 rpm for 5 min to pellet.

5. Wash the cells 2 × with PBS, spinning the cells down at 800 rpm for 5 min to pellet each time.

6. For the final wash, resuspend cells in PBS so that the concentration is 1 × 10^6^ cells/ml.

7. Spot the cells onto 12-well glass slides by pipetting 10 μl of cell suspension into each well, and either dry at room temperature or on a heat block (50°C) until the slides are dry.

8. Store the slides at −20°C for up to 2 months, until needed for immunocytochemistry.

## Anticipated results

### Anticipated results for collagenase dissociation method

Following the temporary culture step, the structure of granulosa cells resembles cultured fibroblasts (Fig. 2A) (Sadowska *et al.*, 2015; Parvari *et al.*, 2016), with adherent cytoplasmic projections and visible speckled lysosomes (numerous in early follicles, see Ndiaye *et al.*, 2015). For an average litter of C57BL/6J (between 6 and 8 ovaries), a sufficient yield of granulosa cells can be accomplished, protocol yields (mean ± SD) 1.07 ± 0.7 × 10^6^ total granulosa cells from a PND1 dissociation, with 1.98 ± 1.8 × 10^6^ from PND4 (Table II). The use of additional ovaries increases the potential cell yield in a weak yet positive linear correlation (*r*^2^ = 0.24, *P* = 0.0131), because the enzyme approaches saturation point (Fig. 2B). We typically achieve a yield of at least 70 ng of RNA per 100 000 cells (Table II), with no significant differences (N = 28, *P* = 0.842) between RNA yield in PND1 and PND4 ovaries (Fig. 2C).

**Table II:** Collagenase dissociation collection characteristics by ovarian timepoint.

Age of dissociated ovaries (n = 5)	Granulosa cell yield per dissociation, mean ± SD	RNA yield (ng RNA per 100,000 cells), mean ± SD	RIN score, mean ± SD
PND1	1.07 ± 0.7 × 10^6^	91 ± 94	9.6 ± 0.4
PND4	1.98 ± 1.8 × 10^6^	70 ± 57	10 ± 0

PND, post-natal day; RIN, RNA integrity number.

Thus, for a dissociation using a typical litter size for C57BL6/J mice (giving between 4 and 8 ovaries), ∼1.3 × 10^6^ cells and approximately 845 ng of total RNA can be obtained. The quality of RNA from dissociated granulosa cells is exceptionally pure as determined via quality control instrument Agilent 2100 bioanalyzer, achieving RNA integrity number (RIN) scores above 9 (Table II), making these samples ideal for next-generation RNA sequencing which typically requires RIN scores > 6 (Kukurba and Montgomery, 2015). Differences in RNA yield and quality may be due to many factors including, but not limited to loss of viability of cells obtained after culture, starting cell numbers and losses at each stage, variation in preparation of reagents and buffers. See Table III for a troubleshooting guide.

**Table III:** Table of troubleshooting tips for collagenase dissociation protocol.

Issue	Possible cause(s)	Advice for solution
Ovary tissue not breaking down during enzymatic dissociation step	Enzymatic failure	Ensure reagents stored and used correctly, that solution is completely combined and that the total quantity of tissue is ≤20 mg
	Tissue rigidity	Older ovaries are more fibrous and may require more forceful mechanical agitation when pipetting puncture/tearing with a needle while in the dissociation solution. Additionally, an extra 15-min incubation in dissociation solution for a total 75 min may suffice
Cells adhering to plate after trypsinisation and wash	Insufficient digestion	Check cells before and after 5-minute incubation with trypsin to ensure they have lifted from the plate. Incubate for a further minute if required. Additionally, tapping plate firmly against a solid surface and pipetting directly onto the plate with force may dislodge any rounded cells remaining on the bottom of the plate
Loss of cell viability after overnight culture	Insufficient nutrients	Ensure ovary culture media is kept at 4°C when not in use, and always heated to 37°C before use. Doubling the proportion of FCS in media to 10% may also increase viability
	Overcrowding on plate	Dilute cell suspension before plating and seed multiple plates, or switch seeding cells in a flask (T25 or T75)
Small/no pellet after collection	Cell loss	Ensure pipette tips and serological pipettes are all coated with Hank’s solution before coming into contact with cells. When not in use, store Hank’s solution at 4°C, equilibrate to room temp before use, and do not use following storage exceeding 2 weeks

**Figure 3.**
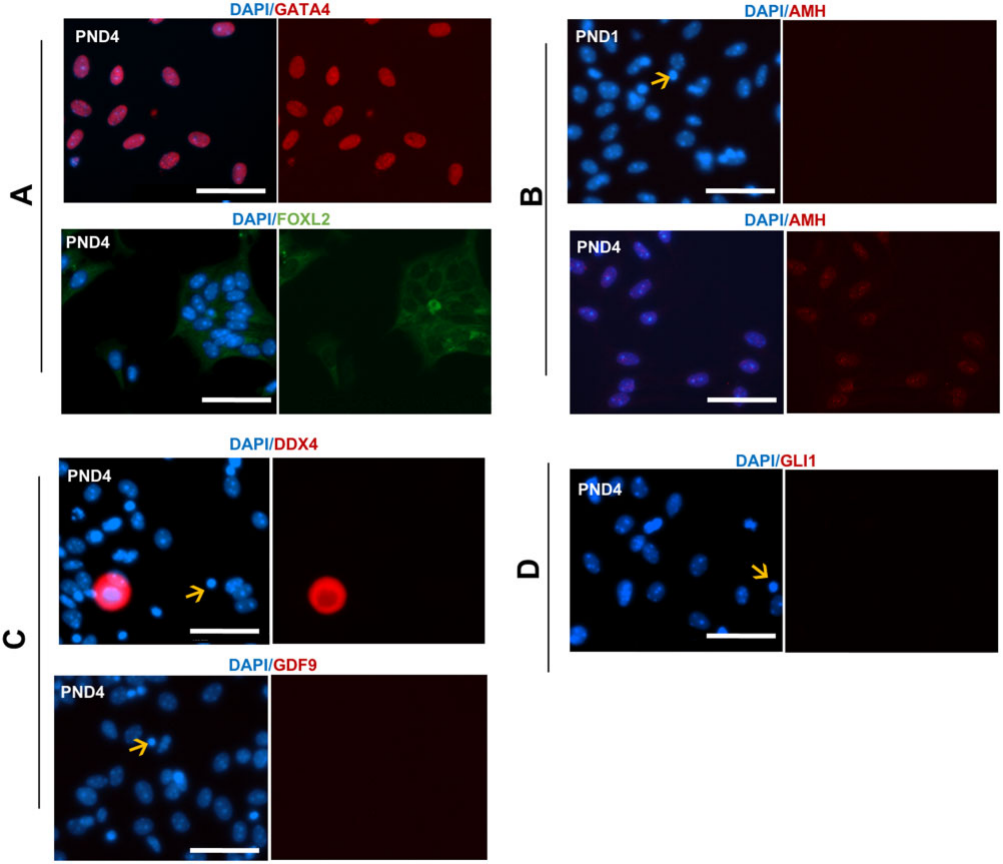
**Fluorescence images for collagenase dissociation method**. (**A**) Immunofluorescent localisation of ovarian cell markers counterstained with blue nuclear marker (4′‐6‐diamidino‐2‐phenylindole (DAPI)) and the granulosa cell markers (GATA4 and FOXL2), (**B**) differentiating granulosa cell marker (AMH) in post-natal day (PND) 1 and PND4 cells dissociated via collagenase, (**C**) oocyte markers (DDX4 and GDF9), and (**D**) theca cell marker (GLI1). Arrows indicates apoptotic cells. All experiments performed in PND1 and PND4 n = 3 for each group, representative images taken at PND4 stage except where indicated. Images taken at 40× magnification, scale bar equivalent to 50 μm.

**Figure 4.**
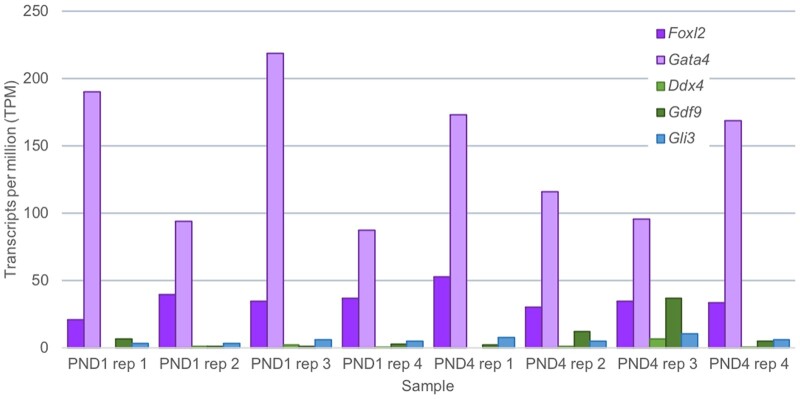
**Gene expression of dissociated cells using the collagenase dissociation method**. Expression of selected ovarian cell-specific markers determined via RNA sequencing of collagenase-dissociated cells originating from both postnatal day (PND) 1 and postnatal day 4 ovaries (n = 4 biological replicates per timepoint). Granulosa cell markers (Foxl2 and Gata4) in purple, oocyte markers (Ddx4 and Gdf9) in green, and theca cell marker (Gli3) in blue. TPM stands for transcripts per million mapped reads and refers to the gene expression value generated via RNA sequencing.

The purity of cells obtained from performing this dissociation can be determined by immunofluorescent detection of a range of markers indicative of ovarian cell types (see Appendix 1 for experimental conditions). Through staining for the granulosa cell markers GATA4 and FOXL2 (Pisarska *et al.*, 2011; Efimenko *et al.*, 2013), the majority of cells post-culture were validated as granulosa cells (Fig. 3A). It was possible to distinguish primordial and primary granulosa cell populations via localisation of AMH, which is expressed in growing granulosa cells from the primary to small antral stage (Visser *et al.*, 2006); thus, it is expressed in cells dissociated from PND4 ovaries, yet not expressed in cells originating from PND1 ovaries (Fig. 3B). In PND4 dissociated cells, markers for oocytes (GDF9 and DDX4; Aaltonen *et al.*, 1999; Raz, 2000) indicated very minimal contamination (Fig. 3C) at a proportion of 0.4% compared to granulosa-positive cells (99.6%) from counts of 300 cells repeated across n = 4 biological replicates (*P* < 0.001). This was mirrored in counts from PND1 dissociated cells, where 0.25% of GDF9 and DDX4 positive cells were identified compared to 99.75% of GATA4 positive granulosa cells from counts of 300 cells repeated across n = 4 biological replicates (*P* < 0.001). Confirming the isolation of a pure granulosa cell population, there was no expression of theca cell marker, GLI1 (Fig. 3D), which is to be expected as theca cells are not observed in the neonatal ovary, but appear once follicles have two or more layers of granulosa cells (Young and McNeilly, 2010), which is not until around PND6 in the mouse. It was observed that following overnight culture, there is some loss in viability of the cells as identified by apoptotic cells with condensed nuclei (denoted by arrows in Fig. 3). These results were confirmed at the gene level by RNA sequencing (see Appendix 1 for method). While a culture step has the capacity to alter the physiology and gene expression of the cell, RNA transcripts of dissociated cells after culture reveal classic granulosa cell expression (*Foxl2 and Gata4*) compared to very minimal or no detection of other ovarian cell markers, specifically oocytes (*Ddx4*, *Gdf9*) and theca cells (*Gli3*) (Fig. 4). In summary, the described technique is an efficient way to collect mass quantities of live granulosa cells from primordial, activating, and primary follicles for downstream functional analyses without the need for manual cell sorting.

### Anticipated results for papain dissociation method

This protocol has been optimised for the dissociation of one ovary per replicate to minimise the need for large amounts of tissue. From one single representative ovary at E18.5, 6.49 ± 2.0 × 10^4^ total cells can be retrieved (Table IV). The average viability of these cells (determined by Trypan Blue staining) is 75% ± 0.1, suitable for any subsequent analyses which require live cells (Table IV). The number of cells obtained increases with increasing ovarian age; 1.25 ± 0.7 × 10^5^ total cells can be retrieved from one single ovary at PND8, with a high cell viability 85% ± 0.0 (Table IV). In the adult ovary, represented by 12 weeks in this manuscript, 3.00 ± 1.0 × 10^5^ total cells are present following dissociation, with a high cell viability of 67% ± 0.1 (Table IV). We typically achieve a yield of 81 ng of RNA per 100 000 cells at E18.5, 78 ng of RNA per 100 000 cells at PND8 and 83 ng of RNA per 100 000 cells at 12 weeks, although this number is variable (Table IV). Variability can be due to a number of factors, the main ones listed in the troubleshooting guide in Table V. RNA integrity was confirmed as high quality by using the Agilent TapeStation instrument, with RIN above 9 for all three timepoints (Table V).

**Table IV:** Papain dissociation collection characteristics by ovarian timepoint (N = 4).

Age of ovary	Cell yield from one ovary, mean ± SD	Cell viability from one ovary, mean ± SD	RNA yield (ng RNA per 100,000 cells), mean ± SD	RIN score, mean ± SD
E18.5	6.49 ± 2.0 × 10^4^	75% ± 0.1	81 ± 49	9.5 ± 0.9
PND8	1.25 ± 0.7 × 10^5^	85% ± 0.0	78 ± 25	9.7 ± 0.3
12WKS	3.00 ± 1.0 × 10^5^	67% ± 0.1	83 ± 43	9.5 ± 0.4

PND, post-natal day; RIN, RNA Integrity number; WKS, weeks.

**Table V:** Table of troubleshooting tips for papain dissociation protocol.

Issue	Possible cause(s)	Advice for solution
Poor tissue dissociation	Enzymatic failure	Ensure the papain enzyme is pre-warmed to 37°C prior to use, and make sure the enzyme is in date. Use freshly prepared papain in HBSS for each dissociation.
	Tissue rigidity	Increase the frequency of the manual dissociation and pipette up and down with 5-mL glass serological pipettes that have been pre-coated with HBSS solution. For older ovaries, the tissue should be minced with a scalpel blade or mincing scissors prior to dissociation. Perform mincing in a glass dish and transfer the minced ovary using a 5-ml glass serological pipette.
Loss of cell viability during the dissociation	Excessive tissue dissociation	Decrease the frequency of the manual dissociation and pipette up and down with 5-ml glass serological pipettes that have been pre-coated with HBSS solution. Decrease the concentration of papain used by 5% to ensure cell viability is maintained. Perform all dissociation steps following the enzymatic dissociation at room temperature, and once completed, immediately snap freeze or fix cells for subsequent procedures.
Cell clumping/a failure to get a single cell suspension	Insufficient tissue dissociation	Increase the frequency of the manual dissociation and pipette up and down with 5-ml glass serological pipettes that have been pre-coated with HBSS solution. Increase the percentage of BSA to 0.05% to prevent cell clumping.
Small/no pellet after dissociation	Cell loss	Ensure pipette tips and serological pipettes are all coated with Hank’s solution before coming into contact with cells. Gently remove the supernatant from the cell pellets after the centrifugations, rather than tipping the tube. This is most effectively done by pipetting off the liquid. When possible, pellet the cells in 15-mL tubes and avoid using round-bottom tubes in the centrifugation steps.

**Figure 5.**
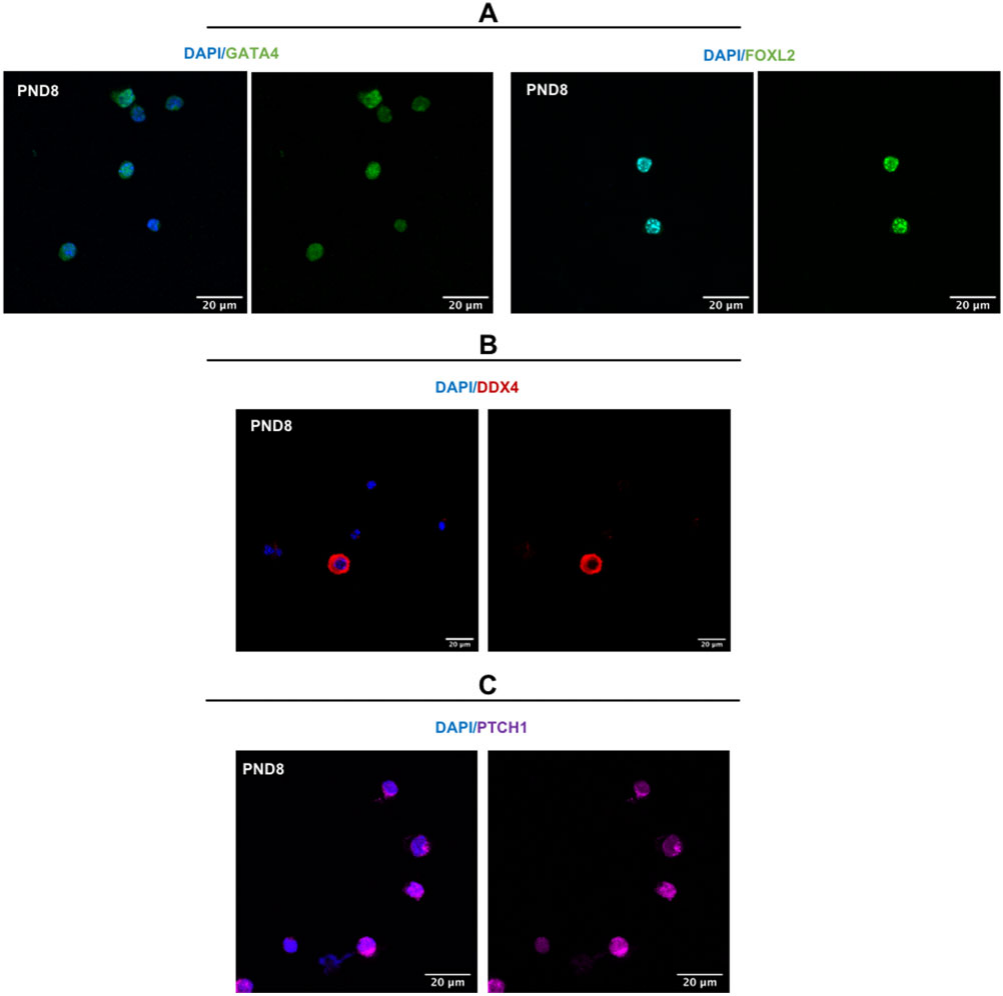
**Fluorescence images for papain dissociation method**. Immunofluorescent localisation of ovarian cell markers counterstained with blue nuclear marker (4′‐6‐diamidino‐2‐phenylindole (DAPI)): (**A**) granulosa cell markers (GATA4 and FOXL2), (**B**) oocyte marker (DDX4), and (**C**) theca cell marker (PTCH1). All experiments performed on dissociated ovarian cells from PND8 ovary (n = 3), and representative images shown. Images taken at 60× magnification, scale bar 20 μm.

**Figure 6.**
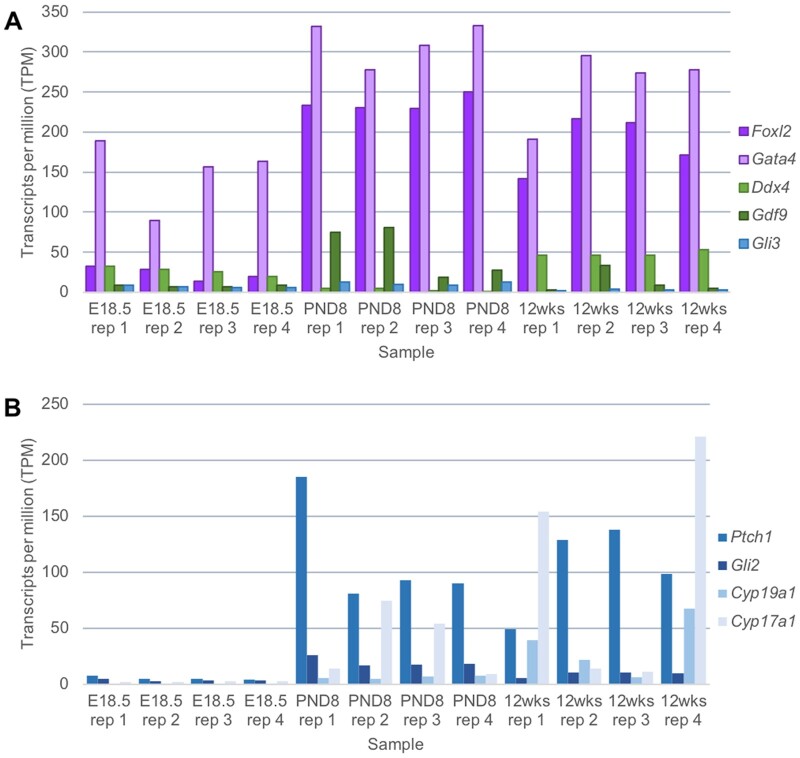
**Gene expression of dissociated cells using the papain dissociation method**. (**A**) Expression of selected ovarian cell-specific markers determined via RNA sequencing of papain-dissociated cells originating from embryonic day 18.5, postnatal Day 8 and postnatal Day 12 week-old ovaries (n = 4 samples per group). Granulosa cell markers (*Foxl2* and *Gata4*) in purple, Oocyte markers (*Ddx4* and *Gdf9*) in green, and theca cell marker (*Gli3*) in blue. (**B**) Expression of selected theca cell-specific markers (*Ptch1, Gli2, Cyp19a1*, and *Cyp17a1*) determined via RNA sequencing of papain-dissociated cells originating from both embryonic Day 18.5, postnatal Day 8 and postnatal Day 12 week-old ovaries (n = 4 samples per group). TPM stands for transcripts per million mapped reads and refers to the gene expression value generated via RNA sequencing.

To determine the percentages of cell types isolated from the PND8 ovary using the papain dissociation protocol, we performed immunofluorescence for several protein markers including the granulosa cell markers GATA4 (Efimenko *et al.*, 2013) and FOXL2 (Schmidt *et al.*, 2004), granulosa cell and theca cell marker PTCH1 (Liu *et al.*, 2015) and oocyte marker DDX4 (Zarate-Garcia et al., 2016) and counted positively stained cells to determine the relative proportions of the different cell types (see Appendix 2 for experimental conditions). These were calculated from counts of 200 cells repeated across 3 biological replicates. 84% of the cells stained positively for both GATA4 and FOXL2, indicating the majority of dissociated cells are granulosa cells (Fig. 5A). The use of a 20 µm filter at the end of the papain dissociation protocol removed most germ cells from the cell suspension, with approximately 1% of cells staining positive for DDX4 (Fig. 5B). 9% of the cells were PTCH1 positive and FOXL2 negative, which is indicative of theca cells (Fig. 5C). The proportion of DDX4 positive cells and PTCH1 positive cells in relation to GATA4/FOXL2 positive granulosa cells is time point dependent. At E18.5, 72% of cells stained positive for GATA4/FOXL2, with 1.7% of cells stained with the oocyte marker DDX4 from counts of 200 cells repeated across n = 3 biological replicates. Dissociated cells from this timepoint showed no PTCH1 expression, consistent with a lack of theca cells present in the embryonic mouse ovary (Abedel-Majed *et al.*, 2019). From cells dissociated from the 12 weeks ovary timepoint, 64% of cells stained positive for GATA4/FOXL2, with 1.6% of cells stained with the oocyte marker DDX4. PTCH1 positive cells were more abundant, with 20% of cells expressing both PTCH1 and FOXL2, indicative of theca cells. These results were confirmed at the gene level, by RNA sequencing (see Appendix 2 for methods). High levels of *Foxl2* and *Gata4* transcripts were detected at all three timepoints (E18.5, PND8 and 12 weeks), with lower levels of the oocyte transcripts *Ddx4* and *Gdf9* and theca cell marker *Gli3* (Fig. 6A). We do observe a higher expression of the oocyte transcripts *Ddx4* and *Gdf9* compared with the collagenase method, potentially due to the presence of oocytes smaller than 20 μm passing through the filter into the dissociated cell population, particularly at the E18.5 timepoint. However, upregulation of other theca markers *Ptch1* and *Cyp17a1* were observed at 12wks, when substantial amounts of theca cells are present in the ovary (Fig. 6B) (Young and McNeilly, 2010; Abedel-Majed *et al.*, 2019; Liu *et al.*, 2015; Richards *et al.*, 2018).

Following dissociation, these cells can be used for a variety of experimental techniques, including fixation steps to perform immunofluorescence protocols. This protocol allows for the dissociation of mouse ovarian tissue at numerous timepoints, to capture all somatic cell lineages for detailed analysis and characterisation.

## Conclusion

These two protocols enable the isolation of multiple cell types from various stages of mouse ovarian development. The collagenase dissociation method is an efficient method for collecting a pure granulosa cell population from the neonatal ovary, whereas the papain dissociation protocol captures all the somatic cell types from the ovary at different developmental stages. There are limitations with both described protocols; namely the extended culture time for the collagenase protocol and the presence of small contaminating cells following filtration using the papain protocol. These limitations should be taken into consideration and used to select an appropriate protocol for the subsequent downstream technologies. To date, it is difficult to isolate pure populations of somatic cell types from the developing mouse ovary, due to a lack of markers to label the various heterogenous cell populations. We present these two methods to facilitate further research into identifying novel markers of mouse ovarian cells and the use of suitable antibodies and fluorescently tags for cell sorting applications.

Both protocols have been shown to allow the preparation of high-quality samples with excellent viability for RNA sequencing, with the advantage of producing a single cell suspension necessary for scRNAseq. In summary, these techniques provide an opportunity to explore mouse ovarian cell function at numerous developmental timepoints.

## Data availability

The data underlying this article are available within it and in Appendices.

## Appendix 1. Collagenase dissociation protocol

### Animal details

C57BL/6J mice were supplied by the University of Newcastle Animal Services Unit under ethics approval number A-2018-803. All experiments involving the use of animals were conducted in accordance with the Institutes’ Animal Care and Ethics Committee guidelines in accordance with Australian NHMRC Guidelines on Ethics in Animal Experimentation.

Mice were housed under a controlled lighting regime (16 L: 8 D) at 21–22°C and supplied with food and water *ad libitum*. Neonatal mice (PND1 and PND4) were euthanized by asphyxiation with carbon dioxide, followed by decapitation. Ovaries were collected immediately after euthanasia, and bursa and uterus remnants removed under a dissecting microscope.

### Immunocytochemistry

Granulosa cells fixed in 24-well plates (as described in materials and methods) were blocked for non-specific binding by an incubation of 3%BSA, 10% donkey serum in PBS for 1 h at room temperature. Granulosa cells were probed with antibodies specific for GATA4 (ab84593; Abcam, Cambridge, UK), FOXL2 (ab5096; Abcam), AMH (130233; Abcam), DDX4 (ab13840; Abcam), GDF9 (ab93892; Abcam), and GLI1 (ab92611; Abcam) at a dilution of 1:300. Primary antibodies were visualised using either a donkey anti-goat Alexa 555-conjugated secondary antibody (a21432; Invitrogen) or a goat anti-rabbit Alexa 555-conjugated secondary antibody (a21428; Invitrogen) at a dilution of 1:200; 4′‐6‐diamidino‐2‐phenylindole (DAPI) was used as a nuclear counterstain. Slides were imaged using the EVOS FL (AMF4300; Thermofisher).

### RNA extraction and quantity

Extractions for granulosa cell RNA were performed using the RNeasy Plus Micro Kit (Qiagen, Hilden, Germany; cat no. 74034) as per the manufacturer’s instructions. Post-extraction, the synthesis of cDNA was completed using the SuperScript IV VILO system (Invitrogen, Carlsbad, CA, USA; cat no. 11766050). The RNA purity, quality, and concentration was determined using the Agilent Bioanalyser 2100. The samples were prepared for this machine via the Agilent RNA 6000 Nano Assay (5067-1511; Agilent, Palo Alto, CA, USA).

### RNA sequencing

Eight, qRNA-seq libraries were sequenced on the NextSeq 2 × 75 bp high output via Auckland Genomics at the University of Auckland. These RNA samples consisted of two groups of mouse granulosa cells (isolated from postnatal Day 1 and postnatal Day 4), with four replicates each. From the output of the RNA sequencing, the bioinformatics team at Auckland Genomics Facility performed the following analyses. The overall quality of the data was visualized using the program FastqQC (Andrews, 2014). TrimGalore (Lindgreen, 2012), was used to check and remove adapters from the sequences. Sequences with a Phred score less than 30 were trimmed. Reads with a length shorter than 25 base pairs were discarded. Hisat2 (Kim *et al*., 2015; Pertea *et al*., 2016) was used to map the cleaned reads to the mouse transcriptome (grcm38). Output sam files from hisat2 were then sorted by genomic position and converted to bam format using samtools sort (Li *et al*., 2009). To generate expression estimates for each sample, the mapped reads were then assembled into transcripts using StringTie. Differential expression analysis was performed using the R software package Ballgown (Fu *et al.*, 2018). Low abundance transcripts with a variance less than one across the samples were removed. To view the similarity between the samples, the Pearson correlation and distance between the samples were calculated. Differential gene expression between the two treatments were calculated using FPKM values. The data were then sorted by their adjusted p-value (false discovery rate) and absolute log2 fold change values.

## Appendix 2. Papain dissociation protocol

### Animal details

C57BL/6J mice were used in all experiments. All experiments carried out on mice were approved under the UK Animal (Scientific Procedures) Act (Project licence 70/8560). Mice were housed in individually ventilated cages under a controlled lighting regime and supplied with food and water *ad libitum*. Mouse husbandry, breeding, ear biopsies and vaginal plug checks were performed by the Biological Research Facility team at the Francis Crick Institute. The day of vaginal plug was considered as E0.5 from the time of conception and the day of birth was termed P0. For E18.5 collections, the mum was euthanised via cervical dislocation, and the E18.5 embryos were dissected from the uterus and euthanised by decapitation. PND8 females were euthanised by decapitation. Twelve-week-old female mice were euthanised by cervical dislocation. At all three timepoints, ovaries were collected immediately following euthanasia and the ovarian bursa was removed under a dissecting microscope.

### Immunocytochemistry

Granulosa cells fixed on glass slides (as described in Materials and methods) were permeabilised in PBS with 0.01% Triton X-100 for 10 min. The cells were blocked with 10% donkey serum in PBS for 1 h at room temperature. Following blocking, granulosa cells were probed with antibodies specific for GATA4 (ab84593; Abcam), FOXL2 (ab5096; Abcam), DDX4 (ab27591; Abcam) and PTCH1 (ab39266; Abcam). Primary antibodies were visualised using either a donkey anti-goat Alexa 555-conjugated secondary antibody (a21432; Invitrogen), a donkey anti-rabbit Alexa 555-conjugated secondary antibody (a32794; Invitrogen) or a donkey anti-mouse Alexa 488-conjugated secondary antibody (a32766; Invitrogen) at a 1:100 dilution and 4′‐6‐diamidino‐2‐phenylindole (DAPI) was used as a nuclear counterstain. Cells were imaged using the Olympus FV1000 Confocal microscope (Olympus America, Center Valley, PA, USA) with a 60× oil immersion lens.

### RNA extraction and quantity

RNA extractions were performed using the RNeasy Plus Micro Kit (Qiagen, Hilden, Germany; cat no. 74034) as per the manufacturer’s instructions. cDNA synthesis was performed using the Superscipt III Reverse Transcriptase kit (Invitrogen, Carlsbad, CA, USA; cat no. 18080-044). The RNA purity, quality and concentration were determined using the Agilent TapeStation.

### RNA sequencing

Twelve libraries were sequenced on an Illumina HiSeq 4000 machine (Illumina, San Diego, CA, USA). The ‘Trim Galore!’ utility version 0.4.2 was used to remove sequencing adaptors and to quality trim individual reads with the q-parameter set to 20 (https://www.bioinformatics.babraham.ac.uk/projects/trim_galore/). The sequencing reads were then aligned to the mouse genome and transcriptome (Ensembl GRCm38release-89) using RSEM version 1.3.0 (Li and Dewey, 2011) in conjunction with the STAR aligner version 2.5.2 (Dobin *et al.*, 2013). Sequencing quality of individual samples was assessed using FASTQC version 0.11.5 (https://www.bioinformatics.babraham.ac.uk/projects/fastqc/) and RNA-SeQC version 1.1.8 (DeLuca *et al.*, 2012). Differential gene expression was determined using the R-bioconductor package DESeq2 version 1.14.1 (Development RC Team, 2008; Love *et al.*, 2014). Gene set enrichment analysis (GSEA) was conducted as described (Subramanian *et al.*, 2005).
